# Effect of Vitamin E on Oocytes Apoptosis in Nicotine-Treated Mice

**Published:** 2012

**Authors:** Ebrahim Asadi, Mehrdad Jahanshahi, Mohammad Jafar Golalipour

**Affiliations:** 1 *Department of Anatomical Sciences, Golestan University of Medical Sciences, Gorgan, Iran*; 2 *Department of Anatomical Sciences, Golestan University of Medical Sciences, Gorgan, Iran*; 3 *Gorgan Congenital Malformation Research Centre, Department of Anatomical Sciences, Golestan University of Medical Sciences, Gorgan, Iran*

**Keywords:** Antioxidant, Cell death, Nicotine, Oocytes, TUNEL assay

## Abstract

**Objective(s):**

Cigarette and nicotine enhances embryogenesis, fertility, pregnancy loss and ultrastructure alterations of oocyte. This study was performed to determine the effect of daily supplementation of vitamin E on oocytes apoptosis in nicotine-treated mice.

**Materials and Methods:**

In this experimental study, 24 NMARI adult female mice were randomly allocated into four experimental groups. For 30 days, animals in control group (C) were received saline through subcutaneous injection, group I received vitamin E (60 mg/kg/day orally), group II received nicotine (5 mg/kg/day, subcutaneous) and animals of group III received nicotine with vitamin E (60 mg/kg/day orally). After 30 days, the animals were superovulated with PSMG (10 Units) and HCG (10 Units). Next day animals were sacrificed and oocytes were flushed. Collected oocytes were examined through TUNEL assay for the determination of apoptosis through the use of fluorescent microscope.

**Results:**

The number of retrieved oocytes was 139, 148, 97 and 127 in control, experimental group I, II and III, respectively. Nicotine treatment increased apoptosis in oocytes up to 13.4% whereas oocytes apoptosis was 3.6% in controls. Supplementation with vitamin E in nicotine-treated mice reduced the oocytes apoptosis to 5.5%.

**Conclusion:**

This study showed that nicotine exposure (5 mg/kg/day for 30 days) can increase apoptosis in oocytes, and supplementation with vitamin E (60 mg/kg/day orally) can reduce the oocytes apoptosis in nicotine-treated mice.

## Introduction

In recent years, in Europe and USA , the rate of smoking in child bearing age women is increasing (1). Smoking can affect quality of follicle, oocyte maturation, fertility and pregnancy rate (2) smoking can also cause follicles loss and chromosomal anomalies (3, 4).

In addition, lower implantation (IR) and pregnancy rate (PR) in smokers undergoing IVF cycles are reported (5).

Nicotine (C_10 _H_14_N_2_) is one of the main active components of cigarette smoke which can increase the generation of free radicals by acting as a pro-oxidant. Nicotine has adverse effect on follicle growth, number of follicles, thickness of endometrium and uterine glands (6). Also, nicotine has adverse effect on cumulus cells and organization of microtubules and microfilaments during meiosis in oocyte.

Nicotine increases lipid per-oxidation following blocking of anti-oxidant enzyme. This process leads to the formation of free radicals or reactive oxygen species (ROS). Free radicals or ROS can cause damage to the cell membrane and DNA fragmentation (7)*. *Also, oxidative stress results to chromosomal instability and program cell death. Program cell death is known as main mechanism in oocyte death. 

Vitamin E as a lipid-soluble substance has anti-oxidant properties*. *Vitamin E has important role in prevention of lipid production and oxidative stress reaction by scavenging free radicals. With regards to anti-oxidant properties of vitamin E , it can reduce the senile oxidative stress reaction on number and quality of oocytes (8). Vitamin E and its various components reduce the oxidative stress reaction by scavenging free radicals in cells and cell organelles.

 With regard to importance of the number of oocytes in fertility and noticeable increase of cigarette smoking in child bearing age women, this study was done to determine the effect of nicotine on oocytes apoptosis and daily supplementation of vitamin E on oocytes apoptosis in nicotine-treated mice.

## Materials and Methods

Female NMARI mice weighing 20–25 g and aged 6-7 weeks were obtained from the Pasteur Institute, Iran. All procedures were performed with approval from the Animal Ethics Committee of Golestan University of Medical Sciences in Iran. The animals were maintained in a climate-controlled room under a 12 hr alternating light/dark cycle (09:00–21:00 hr in light), 20.1 °C to 21.2 °C temperature, and 50% to 55.5% relative humidity. Dry food pellets and water were provided *ad libitum*. Twenty four female mice were randomly divided to four separate groups of six animals each.Control group (group C) received saline (0.2 ml/day by subcutaneous injection). Animals in vitamin E treated group (I) received vitamin E (T-3251; Sigma) (60 mg/kg/day orally). Nicotine-treated group (group II) received nicotine (N-3876; Sigma) (5 mg/kg/day by subcutaneous injection). Also female mice in nicotine with vitamin E treated group (group III) received nicotine (5 mg/kg/day by subcutaneous injection) with vitamin E (60 mg/kg/day orally). The treatments were scheduled for 30 consecutive days.

***Superovulation protocol***

The animals were superovulated following these schedules. Mice were treated with an intraperitoneal injection of 10 IU pregnant mare's serum gonadotrophin (PMSG; Intervet International B.V., Netherlands). Human chorionic gonadotropin (10 IU; Intervet International B.V., Netherlands) was injected intraperitoneally 48 hr later. The next day the animals were sacrificed by cervical dislocation, oocytes were flushed from each fallopian tube using a hypodermic needle with (αMem) media under a dissecting microscope, and the retrieved cumulus oocytes complexes were examined by TUNEL assay.

***TUNEL method***

The retrieved cumulus oocyte complexes were incubated for 60 min in 2% para-formaldyhed and 2 min in Triton-X solution. TUNEL assay (*in situ* cell death detection kit- Roche Germany) was used to determine failure rate of DNA (DNA damage). Incubation with propidium iodide (P4170; Sigma) solution was used for background staining. Then failure rate of DNA was determined by florescent microscope (BX-51 Nikon–Japan) with excitation wave length in the range of 450-500 nm and detection in rang of 515-565 nm.

***Statistical analysis***

Data was analyzed by one way ANOVA and *Post-hock* tests. A *P-*value less than 0.05 was taken as statistically significant. 

## Result

**Figure 1:**
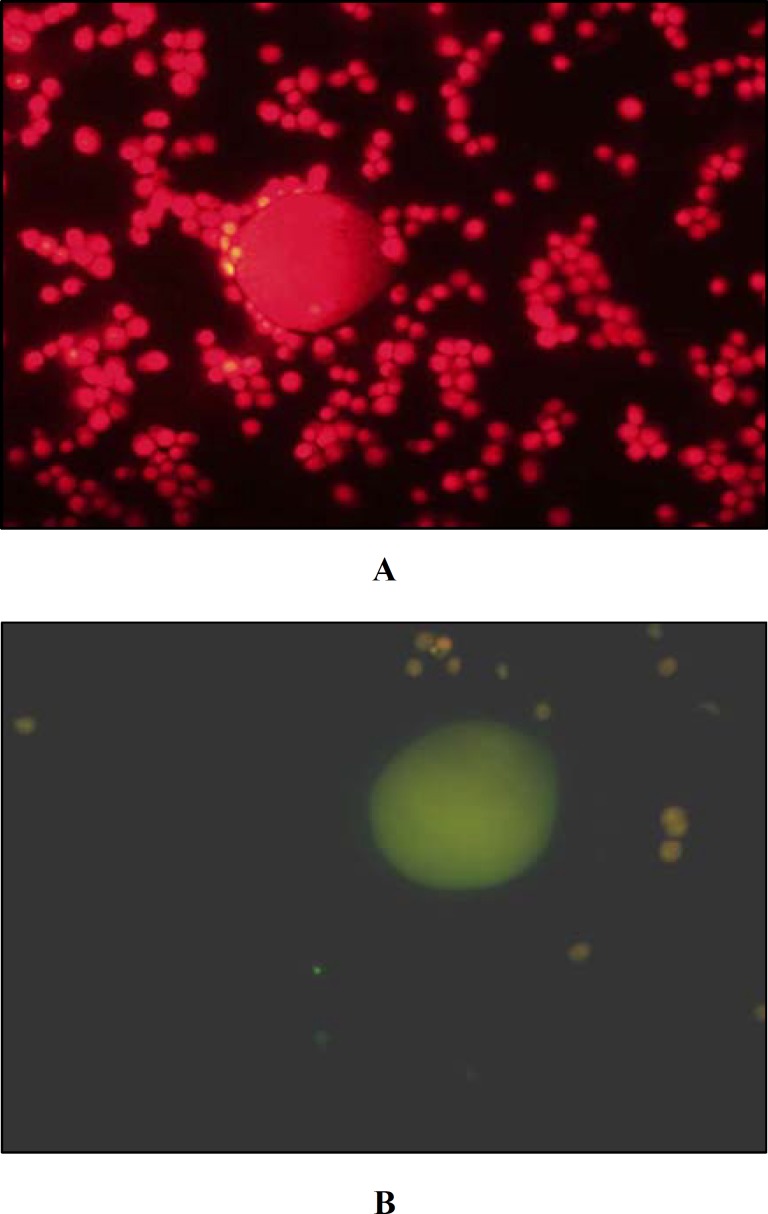
COC were labeled with fluorescein TDT-conjugated dUTP (green channel) and propidium iodide (red channel). (all images 40x). A: Control group B: Nicotine treated oocyte

The number and percent of retrieved and apoptotic oocytes of mice in four experimental groups are depicted in Table 1 and Figure 1. The number and percent of apoptotic oocytes was 5 (3.6%), 13 (13.4%) and 7 (5.5) in control, nicotine treated and nicotine and vitamin E treated mice, respectively. Oocyte apoptosis was significantly increased in nicotine treated mice in comparison with controls (*P*< 0.5). Also oocytes apoptosis in nicotine–vitamin E group was significantly reduced in comparison with nicotine treated mice (*P*< 0.5). There was no significant difference between apoptotic cell number in controls and nicotine-vitamin E group.

## Discussion

This study showed that nicotine exposure increased apoptosis in mice oocytes. Our findings are in accordance with other studies (6, 9, 10-14).

**Table 1:** The number of retrieved oocytes and apoptotic oocytes in control (C), vitamin E (I), nicotine (II) and nicotine with vitamin E (III) groups.

Group	No of Mice	Retrctived oocyte number	Apoptotic oocyte number (%)
C	6	139	5 (3.59%)
I	6	148	6 (4.76%)
II	6	97*	13 (13.4%)*
III	6	127	7 (5.51%) **

*P< 0.05 in nicotine treated (II) in comparison with control (C) and nicotine with vitamin E (III) groups.

**P> 0.05 in nicotine with vitamin E (III) group in comparison with control (C) and vitamin E (I) groups.

Using skin fold chamber model in Syrian golden hamsters and caspase-3 immunohistochemistry of transplanted follicles Bordel study showed that nicotine as a toxic component of cigarette smoke can adversely influence follicular growth by increasing apoptotic cell death (6). Similarly, Mokhtar’s study indicated that nicotine has adverse effect on number and quality of oocyte and fertilization rate in animal model (9).

Furthermore, through Comet assay, Sinko *et al* (2005) showed that smoking is significantly associated with elevated levels of DNA damage in cumulus cells of smoking females which are scheduled for *in vitro* fertilization (10).

Also, Gruslin *et al* study showed that cigarette smoking in pregnant women increased apoptosis in throphoblast cells during first trimester and pro caspase gene expression by TUNEL assay (11).

Through using scanning and transmission electron microscope, Rajikin *et al* showed ultra- structure alterations in oocytes including retained abundant rough endoplasmic reticulum with numerous vesicle and highly dense mitochondria without crista in nicotine treated animals (13). 

Recently, Jennings *et al* (2011) have made use of confocal microscope to determine that oocytes retrieved of mice exposed to smoking had significantly thicker zona pellucida along with shorter and wider meiotic spindles. Furthermore, almost a quarter of oocytes from smoking mice were abnormal as assessed by either errors in chromosomal congression or spindle shape (14). Elsewhere, it causes thickness of ZP and shortness mitotic microtubules and chromosomal disorganization (14).

As one of the main active ingredients of tobacco smoke, nicotine can increase the generation of free radicals by acting as a pro-oxidant. Nicotine also increases the production of reactive oxygen species (ROS) or free radicals, which may lead to oxidative stress in the oocyte (12). Free radical can cause damage to cell membrane, DNA fragmentation (7), microtubules, microfilaments, mitocondria and reticulum endoplacmic (13). By increasing of free radicals nicotine may cause damage to cell membrane, mitochondria and finally DNA fragmentation. These disorders in cells can activate apoptosis processes and cell death. Program cell death is known as main mechanism in oocytes death. 

In our study, oocytes apoptosis in nicotine–vitamin E group were significantly reduced in comparison with nicotine treated mice (*P*< 0.05).

Several studies reported that vitamin E and its compounds can reduce adverse effect of nicotine on oocyte morphology, ultrastructure of oocytes, number and quality of oocytes, zona pellucida and fertilization rate (8, 9, 12, 15).

Rajikin *et al* study showed that administration of g-tocotrienol partially reduced the detrimental effects of nicotine by retaining the smooth boundary of the zona pellucida with the tight perivitelline space and less RER with no visible vesicle and a lower amount of dense mitochondrial matrix (12) .

Mokhtar *et al* study (2008) on Sprague Dawley rats showed that a treatment which makes use of nicotine during pregnancy minimizes the pregnancy outcome to 33.3% while oral supplementation with PVE in nicotine-treated rats augmented the possibility of pregnancy outcome to 83.3%. The percent of embryos developed into two- and four-cell stage in the nicotine plus PVE-treated animals which was more than nicotine-treated rats (9). 

Also, in an experimental animal study, Hassa *et al* indicated that fertilization and cleavage rates were influenced basically in the smoke-exposed female mice population and treatment with vitamin E did affect the fertilization, cleavage, and embryo development rates of smoke-exposed female mice (15).

In an animal model, Train *et al* (8) have shown that oral administration of antioxidant (vitamin E and C) reduced the negative effect of female aging on number and quality of oocytes.

Vitamin E and its various components reduce the oxidative stress reaction by scavenging free radicals in cells and cell organelles (15). In fact vitamin E is the major peroxyl radical scavenger in biological lipid phases such as membranes. Its antioxidant property has been directed toward its ability to chemically act as a lipid-based free radical chain-breaking molecule, hence, preventing lipid peroxidation and protecting the organism in the face of oxidative damage.

## Conclusion

This animal model study showed that nicotine exposure (5 mg/kg/day for 30 days) can increase apoptosis in oocytes and supplementation with vitamin E (60 mg/kg/day orally) can reduce the oocytes apoptosis in nicotine-treated mice.
